# The First Small-Molecule Inhibitors of Members of the Ribonuclease E Family

**DOI:** 10.1038/srep08028

**Published:** 2015-01-26

**Authors:** Louise Kime, Helen A. Vincent, Deena M. A. Gendoo, Stefanie S. Jourdan, Colin W. G. Fishwick, Anastasia J. Callaghan, Kenneth J. McDowall

**Affiliations:** 1 Astbury Centre for Structural Molecular Biology, University of Leeds, Leeds, LS2 9JT, UK; 2 School of Biological Sciences and Institute of Biomedical and Biomolecular Sciences, University of Portsmouth, Portsmouth, PO1 2DY, UK; 3 School of Chemistry, University of Leeds, Leeds, LS2 9JT, UK

## Abstract

The *Escherichia coli* endoribonuclease RNase E is central to the processing and degradation of all types of RNA and as such is a pleotropic regulator of gene expression. It is essential for growth and was one of the first examples of an endonuclease that can recognise the 5′-monophosphorylated ends of RNA thereby increasing the efficiency of many cleavages. Homologues of RNase E can be found in many bacterial families including important pathogens, but no homologues have been identified in humans or animals. RNase E represents a potential target for the development of new antibiotics to combat the growing number of bacteria that are resistant to antibiotics in use currently. Potent small molecule inhibitors that bind the active site of essential enzymes are proving to be a source of potential drug leads and tools to dissect function through chemical genetics. Here we report the use of virtual high-throughput screening to obtain small molecules predicted to bind at sites in the N-terminal catalytic half of RNase E. We show that these compounds are able to bind with specificity and inhibit catalysis of *Escherichia coli* and *Mycobacterium tuberculosis* RNase E and also inhibit the activity of RNase G, a paralogue of RNase E.

The rapid turnover of RNA is central to the regulation of gene expression in all forms of life^1^. It ensures, for example, that translation closely follows programming at the level of transcription. In *Escherichia coli*, a valuable model system, it has been found that a single-strand-specific endoribonuclease called RNase E is required for rapid turnover of mRNA and in addition the processing of many RNAs including those of the translational machinery^2^,^3^. *E. coli* RNase G, a paralogue of RNase E, cooperates with RNase E in the maturation of 16S ribosomal RNA^4^,^5^ and is also involved in the normal degradation of many mRNAs^6^,^7^. RNase E is a potential target for developing new antibiotics, which are increasingly needed given the rising tide of resistance emerging in bacteria of clinical importance. It is essential for the growth of *E. coli*^8^,^9^ and homologues have been found in about half of the bacteria whose genomes have been sequenced, including important pathogens, such as *Salmonella* species, *Yersinia pestis*, *Mycobacterium tuberculosis*, and *Vibrio cholerae*^10^,^11^,^12^,^13^. Furthermore, no homologues of RNase E have been identified in humans or animals.

The N-terminal catalytic half (NTH) of RNase E is a tetramer, which is best described as a dimer of dimers^14^,^15^. Each dimer contributes a pair of equivalent antiparallel channels that include the sites of catalysis. Each channel binds a single-stranded RNA segment and has an adjacent pocket for binding 5′ termini that are both monophosphorylated and unpaired (Fig. 1a–c)^16^. Such termini are found on the downstream products of cleavage and may provide a means of prolonging contact such that additional cleavages can occur^17^. Each single-stranded RNA-binding channel^16^ is formed on one side by a domain that closely resembles the RNA-binding domain of S1^18^ and on the other side by one that resembles the catalytic domain of DNase I^19^. The latter reveals an unexpected link in the evolution of RNA and DNA nucleases. Aspartic acid residues at 303 and 346 in the DNase I domain may act as general bases to activate the hydroxyl group of a water molecule coordinated to the magnesium to attack the scissile phosphate^16^. The scissile phosphodiester bond in the RNA is the point at which cleavage occurs (the bases 3′ to the site of RNA cleavage are defined as +1, +2..., and those 5′ are −1, −2...). The contacts between RNase E and bound RNA are described in the legend to Fig. 1a–c. Recent evidence suggests that simultaneous recognition of two or more unpaired regions may provide a sufficiently stable interaction for efficient cleavage by RNase E to occur^6^,^20^,^21^. However, it is also clear that the efficient cleavage of some substrates requires them to be 5′ monophosphorylated^17^,^22^,^23^. A simple explanation for these observations is that because of structural constraints not all RNAs present a combination of unpaired regions that can cooperate in binding RNase E, but for some of these RNAs a 5′-monophosphorylated end provides an additional foothold for RNase E that allows binding and cleavage of a co-accessible unpaired region^6^,^21^.

The study of RNA processing and decay in *E. coli* has made extensive use of two mutants of RNase E that are temperature sensitive^8^,^9^,^24^,^25^ and have amino acid substitutions in the S1-like domain^26^. However, the interpretation of whether specific steps require the endonucleolytic activity of RNase E has been complicated by reports that residual activity is retained at non-permissive temperatures *in vivo*^27^,^28^ and the observation that for at least one of these mutants aggregation is associated with thermal inactivation *in vitro* (McDowall, K. J. & Stead, J. A., University of Leeds, unpubl. data). The latter has ramifications because RNase E forms the platform for the assembly of the degradosome complex, which includes other components known to play key roles in RNA processing and turnover^29^,^30^. Thus, a method of blocking RNase E activity efficiently without consequence on the structural integrity of the degradosome would be of considerable value in assigning cellular functions to RNase E. Although, for the purpose of studying RNA processing and turnover, it is desirable to be able to block RNase E activity *in vivo*, the ability to do so in cell extracts and preparations of purified components would itself be of considerable value for biochemical studies.

Small molecules that bind within the active sites of essential enzymes have proved to be a source of potent inhibitors and drug leads^31^. In the case of an endoribonuclease, assays can be developed to screen libraries of small molecule inhibitors, that provide a fluorescence output when cleavage of the RNA substrate releases a quencher^32^,^33^. However, when structure(s) of a target protein is available, such as for RNase E, another option is virtual high-throughput screening (VHTS)^34^,^35^. VHTS is a computer-based method that provides a score that reflects the likelihood that individual compounds in a library will be able to bind a target receptor^36^. Herein, we report the combined use of SPROUT^37^ and eHiTS^38^, VHTS computer packages, to identify small molecules that are predicted to target the NTH of *E. coli* RNase E. We also report their activity with regard to binding, inhibition and specificity.

## Results

### Selection of small molecules targeting *E. coli* NTH-RNase E by VHTS

**Figure 1:**
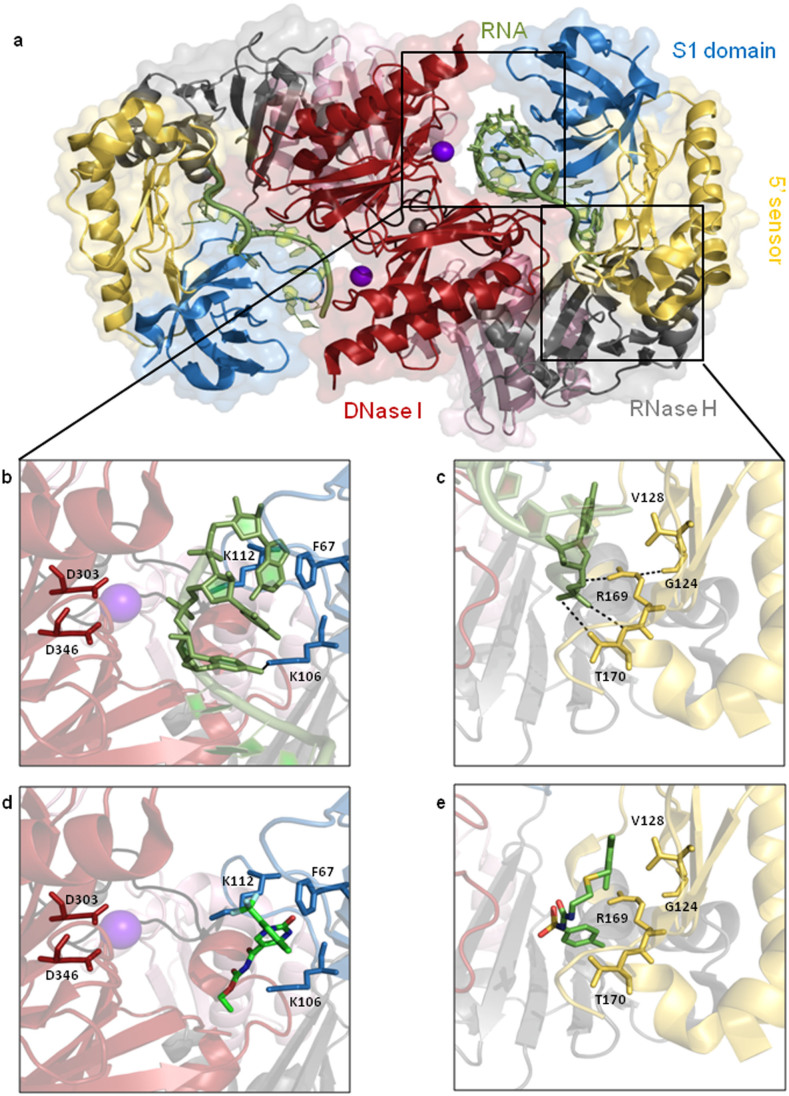
Structure of the RNase E catalytic domain and compound docking. (a) A top elevation of a principal dimer of the N-terminal catalytic domain of *E. coli* RNase E with bound RNA (green). The dimer is shown as a surface representation with the two protomers superimposed as a cartoon diagram. Red, blue, gold and grey colouring identifies the DNase I, S1, 5′ sensor and RNase H domains, respectively. The zinc and magnesium ions are shown as grey and magenta spheres, respectively. (b) The catalytic site. The DNase I side of each of the two channels presents a magnesium ion that is co-ordinated by the carboxylates of aspartic acid residues 303 and 346. The base of the nucleotide at the +2 position relative to the site of RNA cleavage is partitioned into a recess on the surface of the S1 domain. The nucleotide base is held by hydrophobic interactions with a phenylalanine at position 67 and the aliphatic portion of a lysine at position 112. The exocyclic oxygen of the base of the nucleotide immediately 5′ forms a hydrogen bond with a lysine at position 106, also in the S1 domain. (c) The pocket for 5′-monophosphorylated ends contacts both the monophosphate group and the base of the terminal nucleotide. The monophosphate group is hydrogen bonded by the side-chain and peptide amide of a threonine at position 170 and the guanidino group of an arginine at 169: the latter interaction is supported by a hydrogen bond to the peptide backbone of a glycine at position 124. The aromatic ring of the base of the terminal nucleotide is contacted via hydrophobic interaction with the side chain of a valine at 128. (d) The site of catalysis, with predicted docking of compound M5. (e) The 5′-monophosphate binding pocket, with predicted docking of compound P11. The binding of compounds M5 and P11 sterically hinder binding of the RNA molecule.

The site of catalysis and the 5′ monophosphate-binding pocket (Fig. 1b and c) within the single-stranded RNA-binding channel were chosen as targets for VHTS. Two high-resolution X-ray crystal structures of *E. coli* NTH-RNase E as a trapped intermediate with oligoribonucleotide substrates (2BX2 and 2C0B)^16^ were first superimposed using SWISS-PDB Viewer^39^. There were no significant differences in the position of amino-acid residues within the chosen targets of the two X-ray structures (data not shown). Thus, we chose arbitrarily to use the coordinates of the 2C0B entry (3.2 Å resolution) for the 5′ end-binding pocket and the 2BX2 entry (2.8 Å resolution) for the site of catalysis. Within the CAnGAROO module of SPROUT, the 5′ nucleotide of the RNA was used to define a ‘cavity' (*i.e.* the space available for ligand generation) for the 5′ monophosphate-binding pocket, while the nucleotides immediately 5′ and 3′ to the phosphodiester bond that is normally cleaved were used to define a cavity for the site of catalysis. Next, amino acid residues within an envelope that extends 10 Å in radius from the segments of RNA used to derive the cavities were chosen to form the actual receptors for VHTS. 58,833 compounds in the Maybridge database were screened against the ‘5′ monophosphate-binding pocket' and ‘catalytic site' receptors using eHiTS. Compounds with an eHiTS score of ≤−4.0 were then analysed further using SPROUT. Those with log p, eHiTS and SPROUT values of ≤5.0, ≤−4.0 and ≤−4.9, respectively, were viewed - using the SDF file generated by eHiTS - to manually identify those with drug-like properties. The final selection criterion was that compounds should be predicted to form at least one hydrogen bond with RNase E. This was done using the ‘Explore' function of the HIPPO module in SPROUT. This produced a list of 23 compounds against the 5′ end-binding pocket (designated ‘P' compounds) and 10 compounds against the catalytic site (designated ‘M' compounds). Of these only 21 and 9 could be supplied commercially to us, respectively (Supplementary Table S1). The predicted docking of small molecules, described further below, into the catalytic site and 5′ sensor are provided (Fig. 1d and e, respectively). An alignment indicating the residues from *E. coli* RNase E that formed the cavities for docking of small molecules is provided (Supplementary Fig. S1).

### Small molecule binding and inhibition of *E. coli* NTH-RNase E

**Figure 2:**
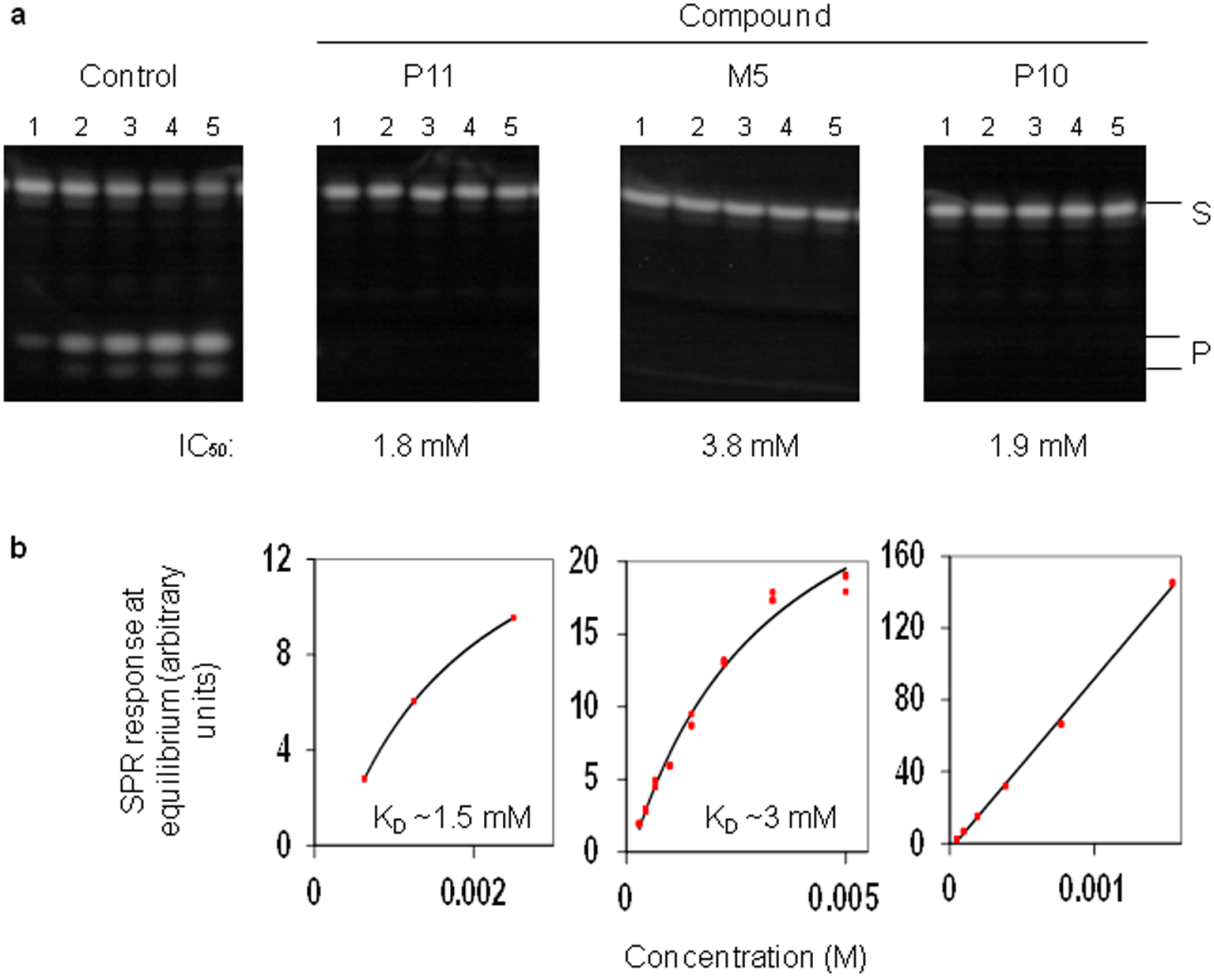
Small molecule binding and inhibition of *E. coli* NTH-RNase E. (a) Discontinuous cleavage assays using the substrate pBR13-fl were carried out as described in Methods. *E. coli* NTH-RNase E was pre-incubated in the absence (control) or presence of the compounds indicated at the top of the panel at final concentrations of 5 mM (P10 and P11) and 20 mM (M5). Lanes numbered 1–5 correspond to samples taken at 0, 2, 5, 10 and 15 min following addition of substrate, respectively. The positions of bands corresponding to substrate (S) and products (P) are indicated on the right. The nominal IC_50_ values for each compound are indicated below each gel. (b) Surface plasmon resonance was used to monitor compound binding to immobilized NTH-RNase E. The concentrations of compound used were 0–2.5 mM for P11, 0–5 mM for M5 and 0–1.5 mM for P10. PBS with 5% DMSO was used as the running buffer. The binding data for each compound concentration at equilibrium is shown (red data points). The steady state fit to the data (black line) gives a K_D_ of ~1.5 mM for P11, ~3 mM for M5 and shows non-specific binding to NTH-RNase E for P10. Compounds P11 and M5 show evidence of beginning to plateau at higher concentrations indicating specific binding, whilst P10 does not.

The ability of the compounds from VHTS to bind and inhibit a recombinant polypeptide of RNase E, used in previous structural and functional studies^14^,^15^,^16^,^40^,^41^, was assayed using Surface Plasmon Resonance (SPR) and a discontinuous cleavage assay, respectively. Eleven compounds were found to bind RNase E and are described below. Seven compounds (M3, M5, M8, M9, P6, P11, and P16) produced binding curves consistent with targeting of specific sites, *i.e*. binding began to plateau at higher concentrations. Of these seven, M5, M8, P6, and P11 clearly inhibited catalysis under the conditions used. Compound M9 also inhibited catalysis but much less efficiently. Three other compounds inhibited RNase E (P10, P14 and P21); however, P10 appeared to bind at sites other than the intended target (*i.e.* binding did not plateau and was seen to be non-specific), while assaying the binding of P14 and P21 was precluded due to their low solubility. The final compound, M2, did not inhibit RNase E under the conditions used and also appeared to bind but at sites other than the intended target. The SPR and inhibition assay results for M5 and P11, which showed the best inhibition of *E. coli* RNase E and bind with specificity, are shown along with P10, providing an example of a compound that inhibits but which appears to bind at sites other than the intended target (Fig. 2). Apparent dissociation constants estimated from the SPR experiments, along with nominal IC_50_ values are included in Table 1.

### Binding and inhibition of *Mycobacterium tuberculosis* RNase E and *E. coli* RNase G

**Figure 3:**
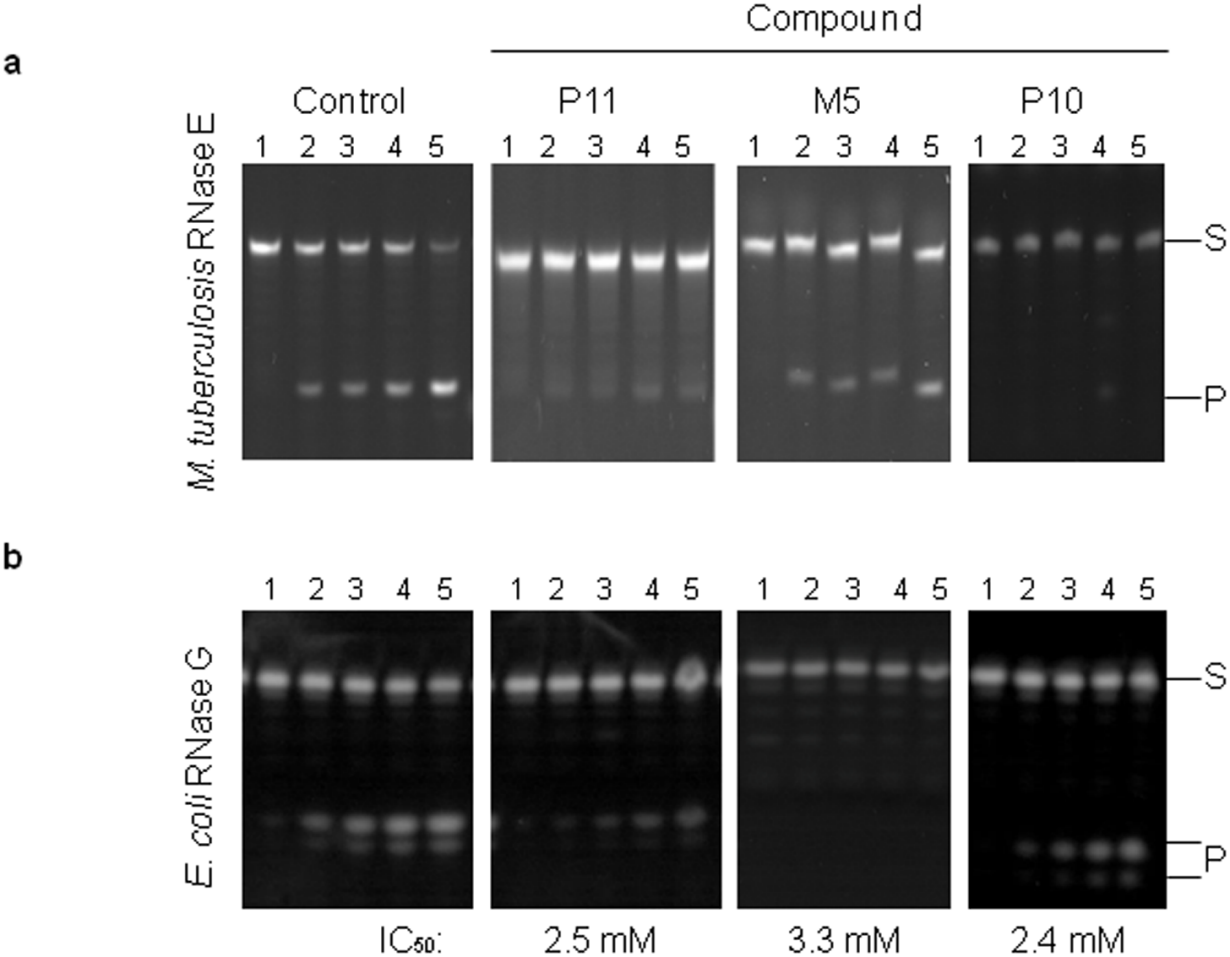
Inhibition of *M. tuberculosis* RNase E and *E. coli* RNase G by small molecules. (a) Denaturing gel analysis of pBR13-fl cleavage by *M. tuberculosis* RNase E. Lanes numbered 1–5 correspond to samples taken at time 0, 2.5, 5, 10 and 30 min, respectively, in the absence of compound (control) and in the presence of 5 mM compound (indicated at the top of the panel). (b) The reaction conditions and labelling for the inhibition of RNase G are as Fig. 2a. Compounds were included in the reactions, as indicated, at final concentrations of 5 mM (P11), 20 mM (M5) and 2.5 mM (P10).

A sequence alignment of the catalytic domain of *E.* coli RNase E with a homologue from *M. tuberculosis* and RNase G, a paralogue of *E. coli* RNase E, show high conservation of residues (Supplementary Fig. S1). The value of compounds that inhibit RNase E, whether as leads for developing new antimicrobials or investigating RNA processing and degradation, is increased if they target homologues in other species as well as the *E. coli* enzyme. Thus, we assayed the ability of a selection of compounds to bind and inhibit RNase E from *M. tuberculosis*, a pathogenic bacterial species of significant clinical relevance^42^,^43^. As found for *E. coli* RNase E, P11 and M5 produced SPR binding curves consistent with specific targeting (data not shown). Compound P11 showed clear inhibition of *M. tuberculosis* RNase E, while M5 inhibits activity but appears to be less efficient than with the *E. coli* enzyme, but most likely reflects the different concentrations of compound used (Fig. 3a and Fig. 2a). It may also reflect subtle differences in the structure of the active site between the *M. tuberculosis* and *E. coli* enzymes and the residues used for docking (Supplementary Fig. S1).

**Figure 4:**
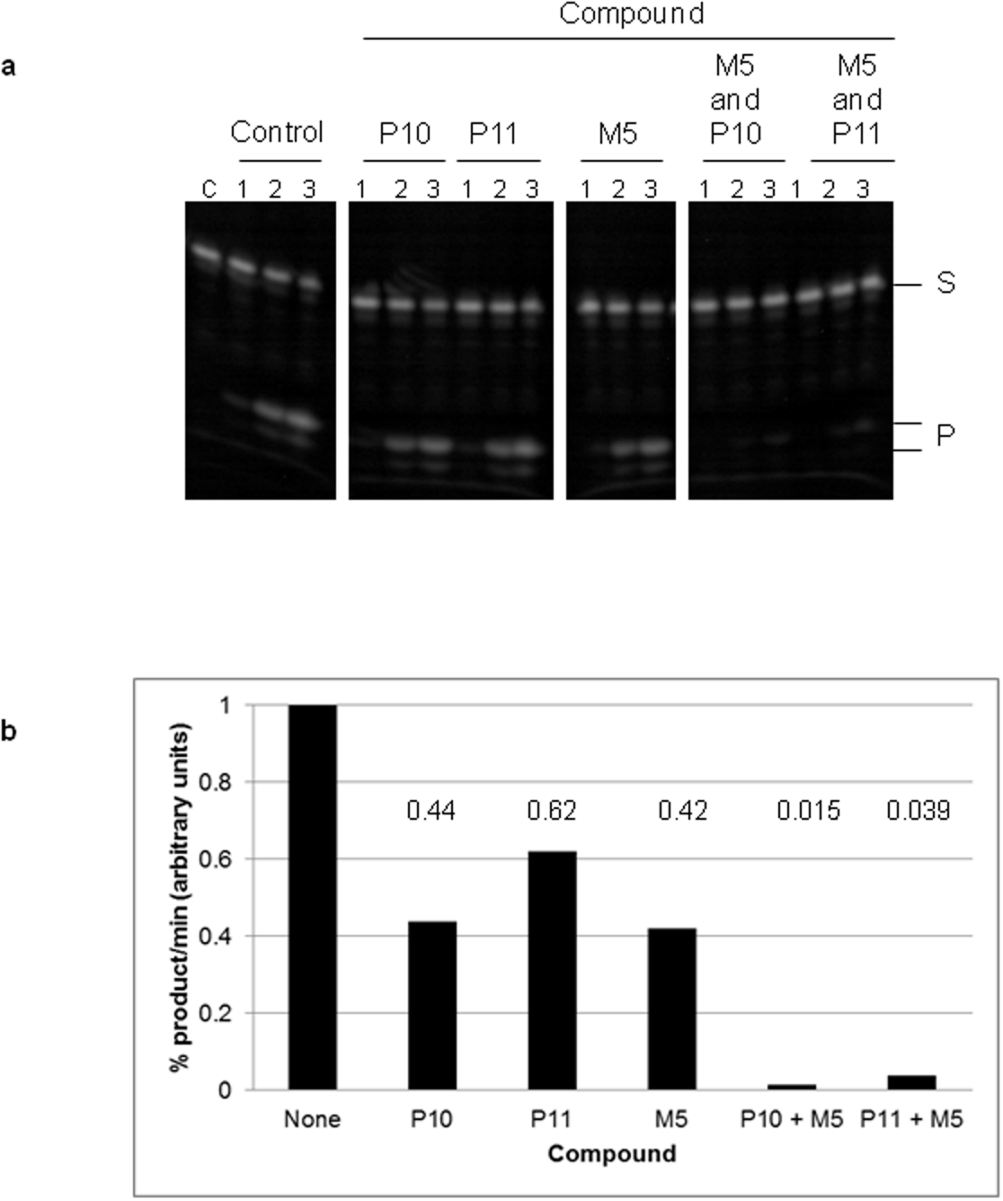
Enhanced inhibition of *E. coli* RNase E by co-incubation with compounds targeted to the 5′ sensor and the catalytic site. (a) The reaction conditions and labelling are as Fig. 2a. Lanes numbered 1–3 correspond to samples taken at 0, 5 and 15 min following addition of substrate, respectively. Lane C contains substrate incubated without enzyme for 15 min. Compounds were included in the reactions at their *E. coli* RNase E IC_50_ concentration; 1.9 mM P10, 1.8 mM P11 and 3.8 mM M5 and were included in combination at the same concentrations for M5 with P10 and M5 with P11. (b) Initial rates were obtained as described in Methods. The initial rate was then normalised to a value of 1 for the reaction containing no compound and represents the product generated in one minute during the initial phase of the reaction. The results are presented as a bar chart.

**Figure 5:**
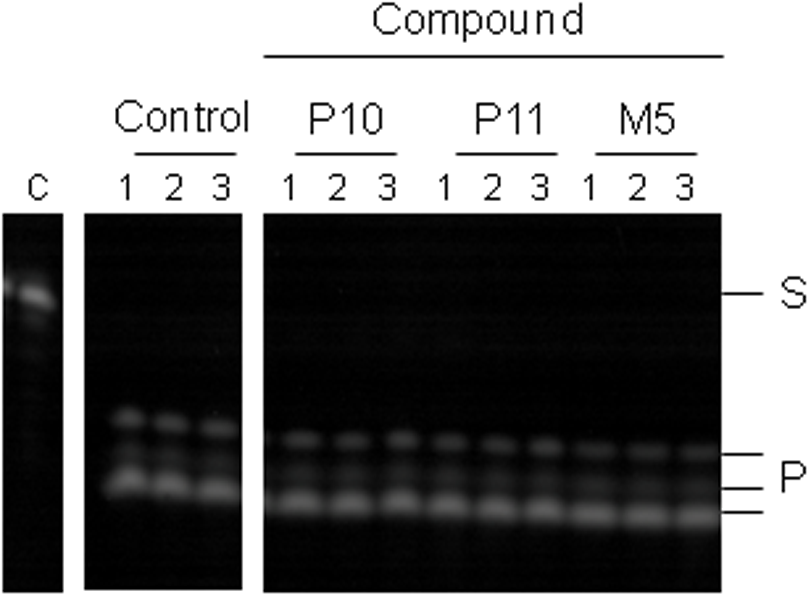
Incubation of small molecules with RNase A. Cleavage assays were performed essentially as described in Methods. RNase A was pre-incubated in the absence (control) or presence of the compounds indicated at the top of the panel at final concentrations of 5 mM (P10 and P11) and 20 mM (M5). Lanes numbered 1–3 correspond to samples taken at 0, 5 and 15 min following addition of substrate, respectively. Lane C contains substrate incubated without enzyme for 15 min. The positions of bands corresponding to substrate (S) and products (P) are indicated on the right.

The compounds shown to inhibit catalysis by *E. coli* RNase E (P6, P10, P11, M5 and M9) were also found to be able to inhibit the activity of RNase G with comparable IC_50_ values (Fig. 3b and Table 1). The ‘P' compounds were slightly less effective with RNase G and, as above, may reflect differences in the 5′-monophosphate binding pocket between RNase E and G and in the residues used for docking (Supplementary Fig. S1). The value of the compounds will be increased further if they can be used in conjunction to target the two different sites (5′ sensor and catalytic site) simultaneously to enhance inhibition of RNase E catalysis. When compound M5 (representing targeting of the catalytic site) was incubated with P11 (targeting of the 5′ sensor) at their IC_50_ concentrations (expect to observe 50% inhibition with the single compound), enhanced inhibition of *E. coli* RNase E was observed to a level of almost 100% (Fig. 4). Whether this enhancement of inhibition is additive or synergistic remains to be determined, but warrants further investigation. To gauge the specificity of inhibition, we found that the compounds shown to inhibit *E. coli* RNase E catalysis did not inhibit the activity of RNase A, an unrelated ribonuclease (Fig. 5).

## Discussion

New and effective antimicrobials are needed to combat the growing number of antibiotic resistant bacteria. The essential enzyme RNase E represents a potential target for the generation of new antimicrobial leads. We have described herein, a method for the selection of small molecule inhibitors against RNase E, which is possible when the structure of the target protein is available. The 5′ sensor and the site of catalysis were chosen as targets in the NTH of *E. coli* RNase E and compounds that were predicted to bind at these sites were selected using VHTS with the combined application of eHiTS^38^ and SPROUT^37^. At least some of the selected compounds were shown to specifically bind and inhibit catalysis by *E. coli* RNase E with K_D_ and IC_50_ values in the low millimolar range (Fig. 2 and Table 1). Furthermore, some of these same compounds can specifically bind and inhibit a homologue from *M. tuberculosis* and inhibit catalysis by *E. coli* RNase G, a paralogue of RNase E (Fig. 3), demonstrating a wider application for these compounds. In addition, inhibition of catalysis is specific for the RNase E family (Fig. 5). Although the K_D_ and IC_50_ values are relatively high, the approach has been validated and the compounds described here can be used as the starting material for developing better, higher affinity inhibitors. It may also be possible to use them in combination (Fig. 4). The best leads from this study for the development of specific small molecule inhibitors of RNase E would include M5, P6 and P11 (compound M8 became unavailable from the Maybridge catalogue during the course of this study).

The hit compounds themselves (Supplementary Table S1), although essentially used in this study as tool compounds in order to probe the ability of small molecules to modulate RNase E function, do indicate the potential ‘druggability' of this system in terms of compliance with the Lipinski guidelines for drug likeness. Specifically, they are either sulfonamido (e.g. P6 and P11) or heterocyclic (e.g. M5), and have molecular weights under 500 (e.g. for P6, P11 and M5, molecular weight = 423, 394, and 420, respectively) with acceptable H-bond donor/acceptor patterns within their structures. The hydrophobic interactions that are usually formed between RNase E and the RNA are mimicked by the inhibitor molecules. For compound M5, the 3-chloro-5-trifluoromethyl pyridine ring is predicted to make hydrophobic interactions with F67 of RNase E and the pyrimidine-2, 4-dione ring is predicted to make hydrophobic interactions with the aliphatic part of K112 analogous to the contacts made to the RNA base immediately 3′ to the cleavage site. There is also the possibility of a hydrogen bond between the ester linkage of the acetylcarbamate and the side chain of K106 analogous to the hydrogen bond formed between the exocyclic oxygen of the base of the nucleotide 5′ to the cleavage site and K106 (Fig. 1d). The trimethylbenzene ring of compound P11 is predicted to make hydrophobic interactions with V128 analogous to the contacts made to the base of the 5′ end nucleotide. There are no obvious hydrogen bonding interactions between RNase E and the tosyl azanecarboxamide group of P11 that are analogous to those formed between T170, R169 and the RNA (Fig. 1e). Modifying this molecule to reintroduce these interactions could improve affinity and inhibition.

In addition to providing leads for new antimicrobials, the inhibitors can be used as part of chemical genetics strategies to dissect the contribution of members of the RNase E family. Finally, the approach described can be extended to identify inhibitors of other families of ribonucleases. For example, it has been recently used in work that will be reported elsewhere to select antibacterials that target 3′ to 5′ exonucleases.

## Methods

### Compound-Enzyme inhibition assays

The NTH of *E. coli* RNase E (residues 1–529) and RNase G were purified as described previously^21^,^40^. The compounds were obtained from Maybridge and re-suspended in 100% DMSO. Discontinuous cleavage assays were done using a 3′ fluorescein-labelled, 5′-monophosphorylated version of BR13, a well-characterised substrate of RNase E, with the sequence 5′-GGGACAGU↓AUUUG^44^. *E. coli* NTH-RNase E was pre-incubated with and without the compounds at 37°C for 20 min before the addition of substrate in a buffer containing 25 mM Bis-Tris Propane (pH 8.3), 100 mM NaCl, 15 mM MgCl_2_, 0.1% Triton X-100, 1 mM DTT and 20% DMSO. The enzyme monomer and substrate concentrations at the start of the reaction were 0.5 and 62 nM, respectively. The inhibition assays with RNase G and RNase A (Sigma) were carried out as above. Reaction products were analysed by electrophoresis using a denaturing 15% polyacrylamide, sequencing-type gel. Detection was via a FLA-5100 scanner (Fuji). Substrate and product were quantified using AIDA software (Raytest Isotopenmessgerate GmbH). Initial rates of reaction at increasing compound concentrations were obtained by establishing the slope representing percentage product generated over time during the initial, linear phase of the reaction. IC_50_ values were calculated by plotting the percentage inhibition of initial rate against compound concentration.

For *M. tuberculosis* RNase E (a kind gift from Prof. B. Luisi, University of Cambridge), 1 μM 3′ fluorescently-labelled, 5′ monophosphorylated BR13 was incubated with 50 nM RNase E with and without the compounds in buffer containing 25 mM Tris-HCl (pH 7.5), 100 mM NaCl, 15 mM MgCl_2_, 1 mM DTT and 5% DMSO at room temperature. Reactions were analysed by electrophoresis using denaturing 20% polyacrylamide gels and visualised using a UV transilluminator.

### Binding studies by SPR analysis

*E. coli* and *M. tuberculosis* RNase E were immobilized covalently using amine coupling to the surface of a Biacore CM5 sensor chip (GE Healthcare). Immobilization levels were approximately 1500 RUs. Assessment of compound binding were conducted by injecting varying compound concentrations in PBS containing 5% DMSO at flow rates of 30–90 μl/min for ~60 s over the reference and test flow cells. A Biacore T100 instrument was used and the data collected was reference and buffer subtracted prior to steady state analysis using data fitting functions provided in the Biacore T100 Evaluation Software.

### eHiTS and SPROUT

The eHiTS program has been described by Zsoldos *et al*^38^. Briefly, eHiTS takes compounds from a library and calculates the optimal conformation each of these ligands can adopt in a cavity of a protein target. The eHiTS approach breaks each ligand into rigid fragments and flexible connecting chains and docks each rigid fragment into every possible place in the cavity. A score is calculated for each structure based upon the geometries of the ligand and the complementarity of surface points on the receptor and ligand: complementary surface points receive a positive score, while repulsive surface points receive a negative score. Other factors are also used to calculate the final score including steric clashes, depth of the cavity, solvation, intramolecular interactions in the ligand, and conformational strain energy of the ligand.

SPROUT^37^ is a computer program that generates structures based upon a set of specified constraints. The approach firstly generates a skeleton based upon a set of primary constraints that includes definition of the target site and must satisfy steric and geometric constraints. This is followed by substitution of atoms in the skeleton to generate molecules with the required properties. The CAnGAROO module within SPROUT allows for the definition of potential binding pockets by detecting clefts, defined as a large inward facing area on the surface of the protein. The HIPPO module locates typical donor and acceptor atoms in the protein, intramolecular hydrogen bonds, hydrogen bonding atoms near to the surface of the receptor site and hydrogen bonding regions are computed with tolerances.

## Author Contributions

C.W.G.F. and D.M.A.G. performed the VHTS. H.A.V. and A.J.C. performed the SPR experiments and the analysis of *M. tuberculosis* RNase E. L.K., S.S.J. and D.M.A.G. performed initial inhibition analysis of *E. coli* RNase E by small molecules. L.K. performed IC_50_ analysis and inhibition of RNase G and RNase A. L.K. and K.J.M. wrote the article with comment by others.

## Supplementary Material

Supplementary Information Supplementary information

## Figures and Tables

**Table 1:** Apparent dissociation constants and IC_50_ values of compounds

Compound	Steady State K_D_ value (mM)	Nominal IC_50_ RNase E (mM)	Nominal IC_50_ RNase G (mM)
P6	~1.0	~4.7	~8.9
P10	non-specific	~1.9	~2.4
P11	~1.5	~1.8	~2.5
P16	~0.6	NI	NI
M2	non-specific	NI	NI
M3	~0.6	NI	NI
M5	~3.0	~3.8	~3.3
M8	~3.5	~3.2	ND
M9	~2.0	~18.0	~24.0

A list of the compounds referred to in the text that showed binding and/or inhibition of RNase E catalysis. The K_D_ values given are for binding to *E. coli* RNase E and the IC_50_ values are for inhibition of the *E. coli* enzymes. K_D_ and IC_50_ values were calculated as described in the Methods. ND = not determined. NI = no inhibition (up to 40 mM tested).

## References

[b1] Condon C. (ed.) Molecular Biology of RNA Processing and Decay in Prokaryotes 85 (Academic Press, Elsevier Inc, 2009).

[b2] Arraiano C. M. *et al.* The critical role of RNA processing and degradation in the control of gene expression. FEMS Microbiol. Rev. 34, 883–923 (2010). 20659169 10.1111/j.1574-6976.2010.00242.x

[b3] Carpousis A. J., Luisi B. F. & McDowall K. J. Endonucleolytic Initiation of mRNA Decay in *Escherichia coli*. Prog Mol Biol Trans Sci. 85, 91–135 (2009). 10.1016/S0079-6603(08)00803-9 19215771

[b4] Li Z. W., Pandit S. & Deutscher M. P. RNase G (CafA protein) and RNase E are both required for the 5′ maturation of 16S ribosomal RNA. EMBO J. 18, 2878–2885 (1999). 10329633 10.1093/emboj/18.10.2878 PMC1171368

[b5] Wachi M., Umitsuki G., Shimizu M., Takada A. & Nagai K. Escherichia coli cafA gene encodes a novel RNase, designated as RNase G, involved in processing of the 5′ end of 16S rRNA. Biochem. Biophys. Res. Commun. 259, 483–488 (1999). 10362534 10.1006/bbrc.1999.0806

[b6] Clarke J. E., Kime L., Romero A. D. & McDowall K. J. Direct entry by RNase E is a major pathway for the degradation and processing of RNA in Escherichia coli. Nucleic Acids Res. 42, 11733–11751 (2015). 25237058 10.1093/nar/gku808 PMC4191395

[b7] Lee K., Bernstein J. A. & Cohen S. N. RNase G complementation of rne null mutation identifies functional interrelationships with RNase E in Escherichia coli. Mol. Microbiol. 43, 1445–1456 (2002). 11952897 10.1046/j.1365-2958.2002.02848.x

[b8] Apirion D. & Lassar A. B. Conditional lethal mutant of Escherichia coli which affects processing of ribosomal RNA. J. Biol. Chem. 253, 1738–1742 (1978). 342528

[b9] Kuwano M. *et al.* Gene affecting longevity of messenger RNA: Mutant of Escherichia coli with altered messenger RNA stability. Mol. Gen. Genet. 154, 279–285 (1977). 337107 10.1007/BF00571283

[b10] Condon C. & Putzer H. The phylogenetic distribution of bacterial ribonucleases. Nucleic Acids Res. 30, 5339–5346 (2002). 12490701 10.1093/nar/gkf691 PMC140075

[b11] Danchin A. A phylogenetic view of bacterial ribonucleases. Molecular Biology of RNA Processing and Decay in Prokaryotes. Condon, C. (ed.) 85, 1–41 (Academic Press, Elsevier Inc, 2009). 10.1016/S0079-6603(08)00801-5 19215769

[b12] Kaberdin V. R., Singh D. & Lin-Chao S. Composition and conservation of the mRNA-degrading machinery in bacteria. J. Biomed. Sci. 18, 23 (2011). 21418661 10.1186/1423-0127-18-23 PMC3071783

[b13] Khemici V., Poljak L., Luisi B. F. & Carpousis A. J. The RNase E of Escherichia coli is a membrane-binding protein. Mol. Microbiol. 70, 799–813 (2008). 18976283 10.1111/j.1365-2958.2008.06454.x PMC7610891

[b14] Callaghan A. J. *et al.* Quaternary structure and catalytic activity of the Escherichia coli ribonuclease E amino-terminal catalytic domain. Biochemistry 42, 13848–13855 (2003). 14636052 10.1021/bi0351099

[b15] Callaghan A. J. *et al.* “Zn-Link”: A metal-sharing interface that organizes the quaternary structure and catalytic site of the endoribonuclease, RNase E. Biochemistry 44, 4667–4675 (2005). 15779893 10.1021/bi0478244

[b16] Callaghan A. J. *et al.* Structure of Escherichia coli RNase E catalytic domain and implications for RNA turnover. Nature 437, 1187–1191 (2005). 16237448 10.1038/nature04084

[b17] Mackie G. A. Ribonuclease E is a 5′-end-dependent endonuclease. Nature 395, 720–723 (1998). 9790196 10.1038/27246

[b18] Subramanian A. R. Structure and Functions of Ribosomal Protein-S1. Prog. Nucleic Acid Res. Mol. Biol. 28, 101–142 (1983). 6348874 10.1016/s0079-6603(08)60085-9

[b19] Suck D. & Oefner C. Structure of DNase I at 2.0 A resolution suggests a mechanism for binding to and cutting DNA. Nature 321, 620–625 (1986). 3713845 10.1038/321620a0

[b20] Kime L., Jourdan S. S., Stead J. A., Hidalgo-Sastre A. & McDowall K. J. Rapid cleavage of RNA by RNase E in the absence of 5′ monophosphate stimulation. Mol. Microbiol. 76, 590–604 (2010). 19889093 10.1111/j.1365-2958.2009.06935.x PMC2948425

[b21] Kime L., Clarke J. E., Romero A. D., Grasby J. A. & McDowall K. J. Adjacent single-stranded regions mediate processing of tRNA precursors by RNase E direct entry. Nucleic Acids Res. 42, 4577–4589 (2014). 24452799 10.1093/nar/gkt1403 PMC3985628

[b22] Garrey S. M. *et al.* Substrate binding and active site residues in RNases E and G: role of the 5′ sensor. J. Biol. Chem. 284, 31843–31850 (2009). 19778900 10.1074/jbc.M109.063263 PMC2797255

[b23] Walsh A. P. *et al.* Cleavage of poly(A) tails on the 3′ end of RNA by ribonuclease E of Escherichia coli. Nucleic Acids Res. 29, 1864–1871 (2001). 11328869 10.1093/nar/29.9.1864 PMC37249

[b24] Ghora B. K. & Apirion D. 5S ribosomal RNA is contained within a 25S ribosomal RNA that accumulates in mutants of Escherichia coli defective in processing of ribosomal RNA. J. Mol. Biol. 127, 507–513 (1979). 372546 10.1016/0022-2836(79)90234-1

[b25] Ono M. & Kuwano M. Conditional lethal mutation in an Escherichia coli strain with a longer chemical lifetime of messenger RNA. J. Mol. Biol. 129, 343–357 (1979). 110942 10.1016/0022-2836(79)90500-x

[b26] McDowall K. J., Hernandez R. G., Lin-Chao S. & Cohen S. N. The ams-1 and rne-3071 temperature-sensitive mutations in the *ams* gene are in close proximity to each other and cause substitutions within a domain that resembles a product of the Escherichia coli mre locus. J. Bacteriol. 175, 4245–4249 (1993). 8320240 10.1128/jb.175.13.4245-4249.1993 PMC204856

[b27] Ow M. C. *et al.* RNase E levels in Escherichia coli are controlled by a complex regulatory system that involves transcription of the *rne* gene from three promoters. Mol. Microbiol. 43, 159–171 (2002). 11849544 10.1046/j.1365-2958.2002.02726.x

[b28] Mohanty B. K. & Kushner S. R. Rho-independent transcription terminators inhibit RNase P processing of the secG leuU and metT tRNA polycistronic transcripts in Escherichia coli. Nucleic Acids Res. 36, 364–375 (2008). 18033800 10.1093/nar/gkm991 PMC2241853

[b29] Carpousis A. J. The RNA degradosome of Escherichia coli: An mRNA-degrading machine assembled on RNase E. *Annu*. Rev. Microbiol. 61, 71–87 (2007). 10.1146/annurev.micro.61.080706.093440 17447862

[b30] Marcaida M. J., DePristo M. A., Chandran V., Carpousis A. J. & Luisi B. F. The RNA degradosome: Life in the fast lane of adaptive molecular evolution. Trends Biochem. Sci. 31, 359–365 (2006). 16766188 10.1016/j.tibs.2006.05.005

[b31] Bleicher K. H., Bohm H. J., Muller K. & Alanine A. I. Hit and lead generation: Beyond high-throughput screening. Nat. Rev. Drug. Discov. 2, 369–378 (2003). 12750740 10.1038/nrd1086

[b32] Zelenko O. *et al.* A novel fluorogenic substrate for ribonucleases: Synthesis and enzymatic characterization. Nucleic Acids Res. 22, 2731–2739 (1994). 8052528 10.1093/nar/22.14.2731 PMC308241

[b33] Jiang X. Q. & Belasco J. G. Catalytic activation of multimeric RNase E and RNase G by 5′-monophosphorylated RNA. Proc. Natl. Acad. Sci. USA 101, 9211–9216 (2004). 15197283 10.1073/pnas.0401382101 PMC438955

[b34] Guner O., Clement O. & Kurogi Y. Pharmacophore modeling and three dimensional database searching for drug design using catalyst: Recent advances. Curr. Med. Chem. 11, 2991–3005 (2004). 15544485 10.2174/0929867043364036

[b35] Seifert M. H. J., Kraus J. & Kramer B. Virtual high-throughput screening of molecular databases. Curr. Opin. Drug Di. De. 10, 298–307 (2007). 17554856

[b36] Shoichet B. K. Virtual screening of chemical libraries. Nature 432, 862–865 (2004). 15602552 10.1038/nature03197 PMC1360234

[b37] Gillet V. J. *et al.* SPROUT: Recent developments in the de novo design of molecules. J. Chem. Inf. Comput. Sci. 34, 207–217 (1994). 8144711 10.1021/ci00017a027

[b38] Zsoldos Z., Reid D., Simon A., Sadjad S. B. & Johnson A. P. eHiTS: A new fast, exhaustive flexible ligand docking system. J. Mol. Graphics Modell. 26, 198–212 (2007). 10.1016/j.jmgm.2006.06.002 16860582

[b39] Guex N. & Peitsch M. C. SWISS-MODEL and the Swiss-PdbViewer: An environment for comparative protein modeling. Electrophoresis 18, 2714–2723 (1997). 9504803 10.1002/elps.1150181505

[b40] Jourdan S. S. & McDowall K. J. Sensing of 5′ monophosphate by Escherichia coli RNase G can significantly enhance association with RNA and stimulate the decay of functional mRNA transcripts in vivo. Mol. Microbiol. 67, 102–115 (2008). 18078441 10.1111/j.1365-2958.2007.06028.x

[b41] Redko Y. *et al.* Determination of the catalytic parameters of the N-terminal half of Escherichia coli ribonuclease E and the identification of critical functional groups in RNA substrates. J. Biol. Chem. 278, 44001–44008 (2003). 12947103 10.1074/jbc.M306760200

[b42] Dye C. & Williams B. G. The population dynamics and control of tuberculosis. Science 328, 856–861 (2010). 20466923 10.1126/science.1185449

[b43] Koul A., Arnoult E., Lounis N., Guillemont J. & Andries K. The challenge of new drug discovery for tuberculosis. Nature 469, 483–490 (2011). 21270886 10.1038/nature09657

[b44] McDowall K. J., Kaberdin V. R., Wu S. W., Cohen S. N. & Lin-Chao S. Site-specific RNase E cleavage of oligonucleotides and inhibition by stem-loops. Nature 374, 287–290 (1995). 7533896 10.1038/374287a0

