# Supplementary material for: The First Small-Molecule Inhibitors of Members of the Ribonuclease E Family
Source: Sci Rep. 2015 Jan 26;5:8028. doi: 10.1038/srep08028 (PMC4306137; doi:10.1038/srep08028)
Supplement: Supplementary Information [file srep08028-s1.pdf]

SUPPLEMENTARY INFORMATION FOR:

The First Small-Molecule Inhibitors of Members of the Ribonuclease E Family

Louise Kime[1]*, Helen A. Vincent[2], Deena M. A. Gendoo[1], Stefanie S. Jourdan[1], Colin W. G. Fishwick[3], Anastasia J. Callaghan[2] and Kenneth J. McDowall[1]

*Corresponding author: Louise Kime (l.kime@leeds.ac.uk)

[1]Astbury Centre for Structural Molecular Biology, School of Molecular and Cellular Biology, Faculty of Biological Sciences, University of Leeds, Leeds, LS2 9JT, UK

[2]School of Biological Sciences and Institute of Biomedical and Biomolecular Sciences, University of Portsmouth, Portsmouth, PO1 2DY, UK

[3]School of Chemistry, University of Leeds, Leeds, LS2 9JT, UK

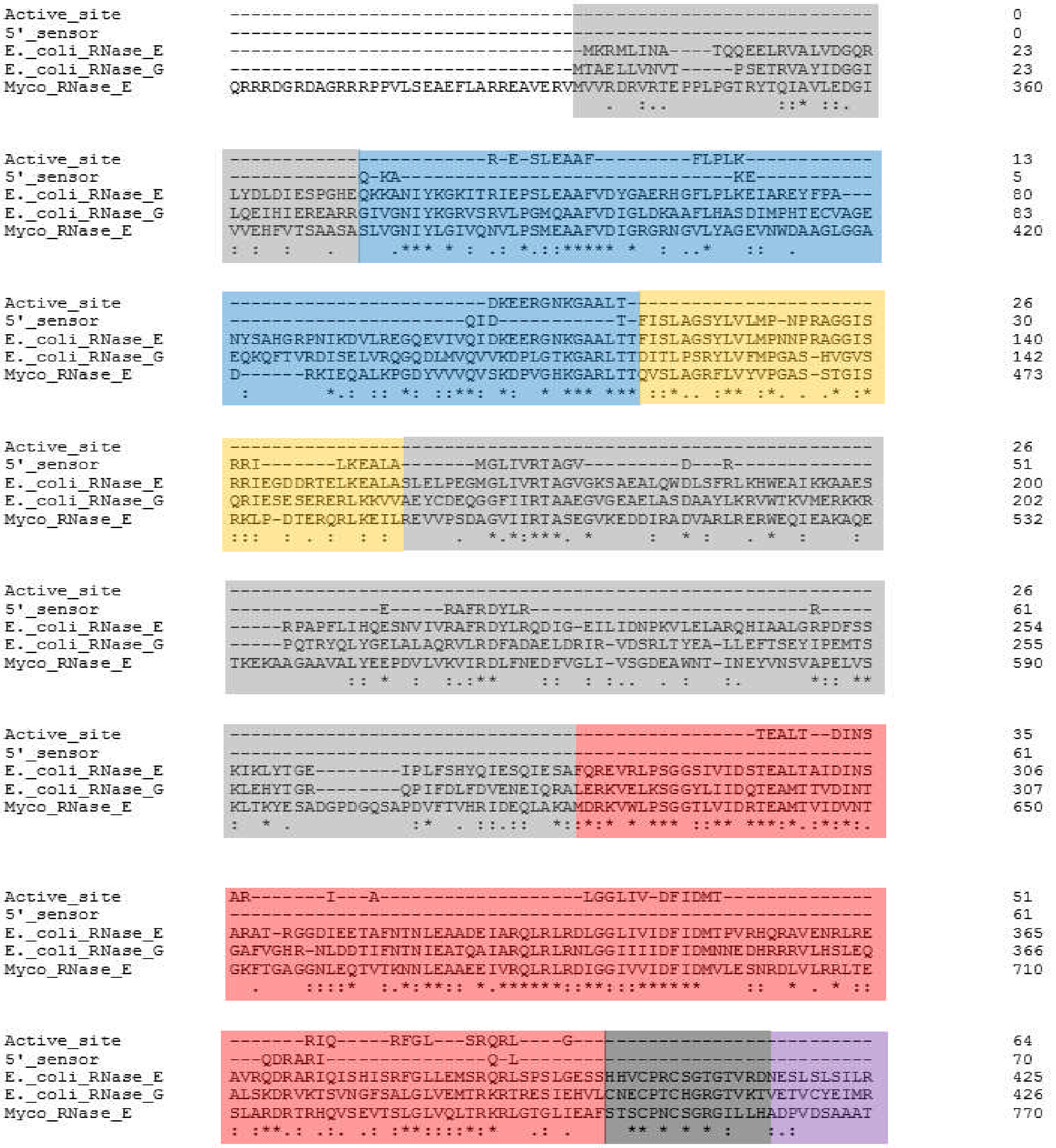

Supplementary Figure S1. Sequence alignment of the RNase E catalytic domain. Sequence alignment of the N-terminal catalytic half of *E. coli* RNase E with *E. coli* RNase G and *M. tuberculosis* (Myco) RNase E. The residues from *E. coli* RNase E that formed the pocket for docking of compounds for the active site and 5' sensor are indicated at the top of each row, respectively. The subdomains RNase H (pale grey), S1 (blue), 5' sensor (gold), DNase I (red), Zn-link (dark grey) and small domain (violet) are shown. The protein sequences were aligned using Clustal Omega.[1] The degree of conservation of the residues is indicated at the bottom of each row: asterisk (*) indicates that the residues are identical; colon (:) indicates conserved substitutions; and period (.) indicates semi-conserved substitutions.

Supplementary Table S1. Compounds identified from docking study

Target site	Name	Maybridge code	Structure	eHiTS	SPROUT
5' phosphate-pocket binders	P1	HTS12494	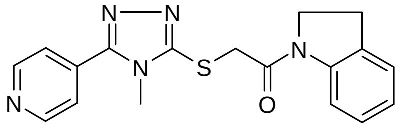	-4.25	-5.52
	P2	HTS11994	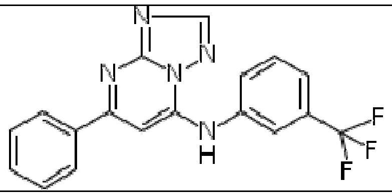	-4.1	-5.64
	P3	HTS09613	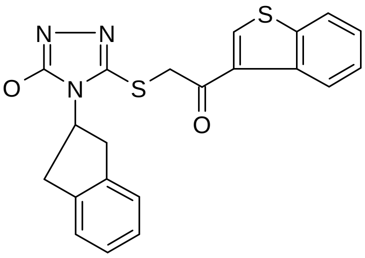	-4.18	-5.95
	P4	HTS02195	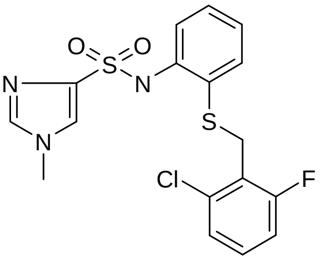	-4.31	-5.19
	P5	HTS01524	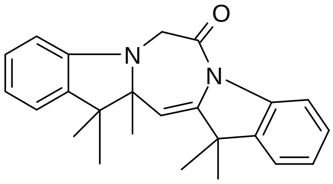	-4.39	-5.45
	P6	HTS01081	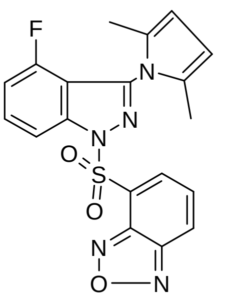	-4.6	-5.35
	P7	DSHS00853	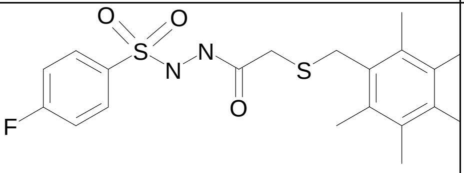	-4.56	-5.12

	P8	CD11294	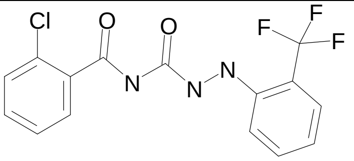	-4.74	-4.93
	P9	CD02581	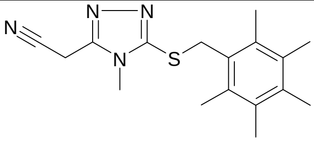	-5.14	-5.41
	P10	AW01130	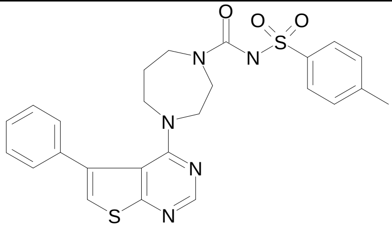	-4.34	-5.38
	P11	SEW06445	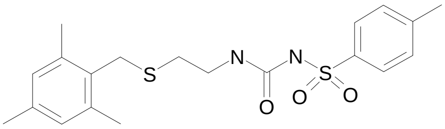	-4.25	-5.49
	P12	RJC01648	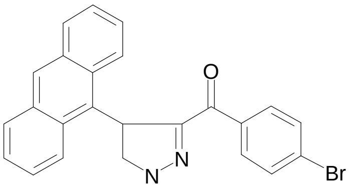	-4.66	-5.41
	P13	RJC00192	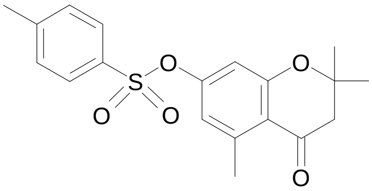	-4.58	-5.68
	P14	RH01661	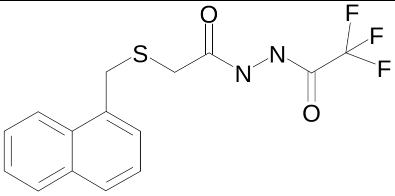	-4.13	-5.52
	P15	RF04853	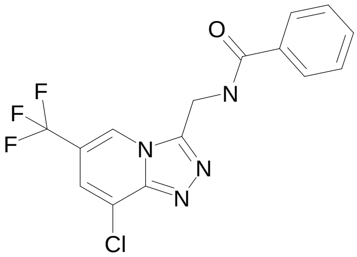	-4.73	-5.19
	P16	KM09759	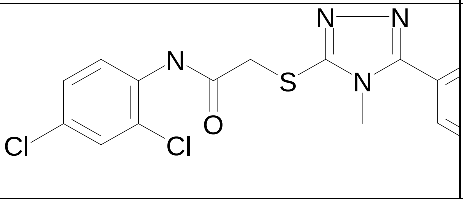	-4.22	-5.93

	P17	KM07797	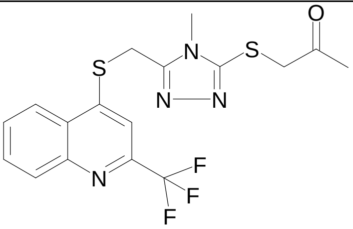	-4.46	-5.03
	P18	KM06506	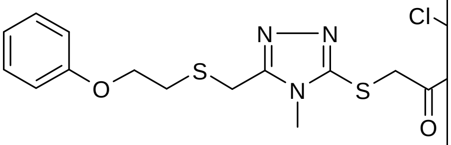	-4.15	-5.56
	P19	JFD01097	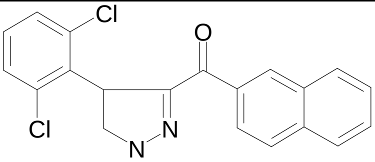	-4.51	-4.90
	P20	HTS12496	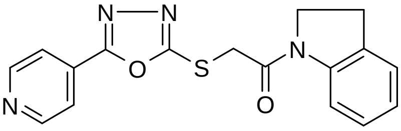	-5.05	-5.52
	P21	S03067	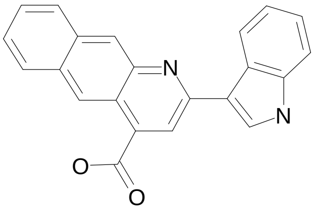	-4.45	-5.29
Catalytic site binders	M1	BTB06821	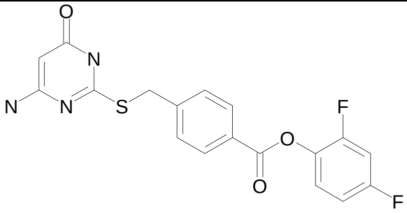	-5.23	-5.59
	M2	CD01377	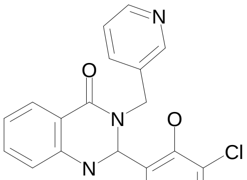	-5.22	-5.40
	M3	KM03247	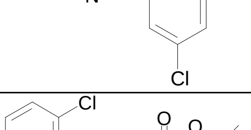	-5.23	-5.49
	M4	KM08139	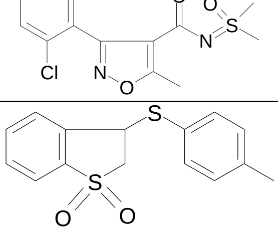	-5.26	-5.64

	M5	KM08782	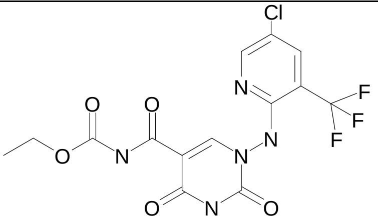	-5.34	-6.43
	M6	NRB00062	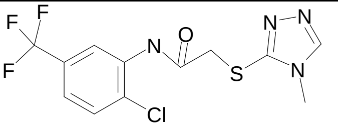	-5.13	-5.46
	M7	SEW06428	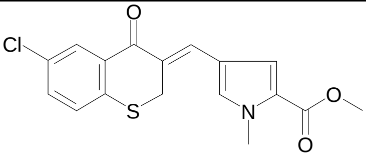	-5.21	-5.75
	M8	SPB02893	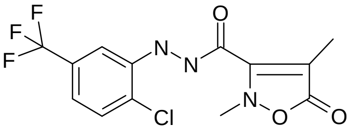	-5.31	-6.39
	M9	SPB03404	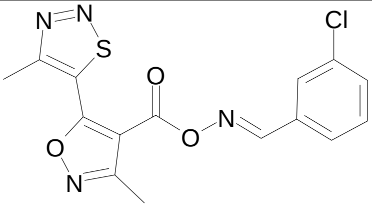	-5.12	-5.83

A list of the compounds available to us generated by VHTS (see main text for details). The compounds are categorised according to targeting of the 5' sensor (P compounds) or the catalytic site (M compounds). The structure of each of the compounds, the Maybridge code and the eHiTS and SPROUT scores are provided.

1 Sievers, F. *et al.* Fast, scalable generation of high-quality protein multiple sequence alignments using Clustal Omega. *Mol. Sys. Biol.* **7**, doi:Artn 539
Doi 10.1038/Msb.2011.75 (2011).
